# ERK1 and ERK2 mitogen-activated protein kinases affect Ras-dependent cell signaling differentially

**DOI:** 10.1186/jbiol38

**Published:** 2006-06-28

**Authors:** Chiara Vantaggiato, Ivan Formentini, Attilio Bondanza, Chiara Bonini, Luigi Naldini, Riccardo Brambilla

**Affiliations:** 1 Istituto Scientifico San Raffaele and Università Vita-Salute San Raffaele, Via Olgettina 58, 20132 Milano, Italy; 2 Current address: Istituto Scientifico E. Medea, 23848 Bosisio Parini, Italy

## Abstract

**Background:**

The mitogen-activated protein (MAP) kinases p44^ERK1 ^and p42^ERK2 ^are crucial components of the regulatory machinery underlying normal and malignant cell proliferation. A currently accepted model maintains that ERK1 and ERK2 are regulated similarly and contribute to intracellular signaling by phosphorylating a largely common subset of substrates, both in the cytosol and in the nucleus.

**Results:**

Here, we show that ablation of ERK1 in mouse embryo fibroblasts and NIH 3T3 cells by gene targeting and RNA interference results in an enhancement of ERK2-dependent signaling and in a significant growth advantage. By contrast, knockdown of ERK2 almost completely abolishes normal and Ras-dependent cell proliferation. Ectopic expression of ERK1 but not of ERK2 in NIH 3T3 cells inhibits oncogenic Ras-mediated proliferation and colony formation. These phenotypes are independent of the kinase activity of ERK1, as expression of a catalytically inactive form of ERK1 is equally effective. Finally, ectopic expression of ERK1 but not ERK2 is sufficient to attenuate Ras-dependent tumor formation in nude mice.

**Conclusion:**

These results reveal an unexpected interplay between ERK1 and ERK2 in transducing Ras-dependent cell signaling and proliferation. Whereas ERK2 seems to have a positive role in controlling normal and Ras-dependent cell proliferation, ERK1 probably affects the overall signaling output of the cell by antagonizing ERK2 activity.

## Background

The small GTPase Ras, its relatives and their effectors are central to the signaling networks that are involved in a variety of regulatory processes in the cell, from proliferation and tumorigenesis to development and synaptic plasticity [1-3]. The signaling cascade involving the Raf, MEK (mitogen-activated protein (MAP) or extracellular signal-regulated (ERK) kinase) and ERK families of kinases is among the best characterized pathways downstream of Ras. This signaling module couples receptor-mediated activation of Ras to cytoplasmic and nuclear events, leading to phosphorylation of key structural and regulatory components [4-8].

Approximately 15% of human cancers contain activating mutations in one of the Ras genes [1,9]. This figure under-represents the actual involvement of Ras pathways in tumorigenesis, however, as other downstream signaling components, such as B-Raf, are frequently found in their oncogenic form in tumors in which Ras is not itself mutated [10]. Importantly, though, induction of missense activating mutations or deletions in regulatory domains might not be the only mechanism leading to deregulation of the Ras-ERK pathway and malignancy. Although there is no evidence so far to suggest that either MEK1/2 or ERK1/2 proteins can become oncogenic in spontaneous tumors, their activity is massively upregulated in several human cancers [11]. For instance, in human leukemia samples, both MEKs and ERKs are often hyperphosphorylated and activated, suggesting a causal relationship between stimulation of the Ras-ERK pathway and tumorigenesis and providing a conceptual framework for potential therapeutic targeting (as reviewed in [12]).

One important aspect of the regulation of the Ras-ERK cascade is the specific, non-redundant role of protein isoforms in this pathway. Gene-targeted and transgenic mouse lines have proved invaluable in determining specific phenotypes associated with most signaling components in the pathway, including lines defective in one of all three Ras proteins (K-ras, N-ras and H-ras), the Raf isoforms c-Raf-1, Raf-A and Raf-B, the MEKs MEK1 and MEK2, the Ras GTPase-activating proteins GAP-1 and NF1, the Ras guanine nucleotide-releasing factors RasGRF1 and RasGRF2, and the adaptor proteins Sos1, Grb2 and Shc [1,4,13-24]. Moreover, for some components of the pathway, such as c-Raf-1 and B-Raf, significant structural differences are the basis not only of their differential regulation, but possibly also of their oncogenic potential [25].

Surprisingly, relatively little is known about possible specific roles for the two major ERK isoforms, ERK1 (p44) and ERK2 (p42). These two proteins are co-expressed in virtually all tissues but with a remarkably variable relative abundance, ERK2 being the predominant isoform in brain and hematopoietic cells [12,26,27]. Given the extensive aminoacid identity between the two molecules and their apparently similar spatio-temporal regulation, the current working model regards them essentially as interchangeable. Nevertheless, important recent evidence suggests that there could be quantitative differences in ERK1 and ERK2 dynamics and that these could have a significant role in their regulation. ERK1-deficient mice are viable, with no obvious compensatory upregulation of ERK2 protein levels but showing a deficit in thymocyte maturation [28]. A recent T-cell-specific knockout of ERK2 further supports a crucial role for MAP-kinase signaling in the immune system [29]. On the other hand, global ERK2-deficient mice die early in development, showing that ERK1 cannot compensate in the embryo for ERK2 [30-32].

One possible interpretation of these data is that although ERK2 is essential for transduction of signals, ERK1 could instead have an accessory role, possibly enabling a fine tuning of ERK2 activity. Two related lines of evidence strongly support the idea that ERK1 acts in a complex manner, at least in certain circumstances, by attenuating ERK2 activity. First, both in fibroblasts and in neurons derived from ERK1-deficient mice, stimulus-dependent activation of ERK2 (but not its basal activity) was found to be significantly upregulated, as revealed by the increased level of ERK2 phosphorylation and immediate-early gene transcription [28,33]. Second, enhancement of ERK2-dependent signaling in the nervous system of the ERK1 mutant mice has been linked to improvement of certain forms of learning and memory [33].

To investigate whether such mechanisms are also implicated in the control of cell proliferation, we examined ERK activation and growth rates both in genetically altered mouse fibroblasts and using RNA interference (RNAi) technology [34-36].

## Results and discussion

### Enhancement of ERK2 signaling in ERK1 mutant fibroblasts provides a significant growth advantage

Our own previous work [33] has shown that in primary neurons of the central nervous system, neurotransmitter stimulation results in a significant hyperactivation of ERK2 in the absence of ERK1. On the basis of these findings, we proposed a competition model between ERK1 and ERK2 in their interaction with the upstream kinase MEK. According to this model, we speculated that in the absence of ERK1, the pool of ERK2 molecules could be more efficiently activated, resulting in an increased downstream transmission of the signal [33].

**Figure 1:**
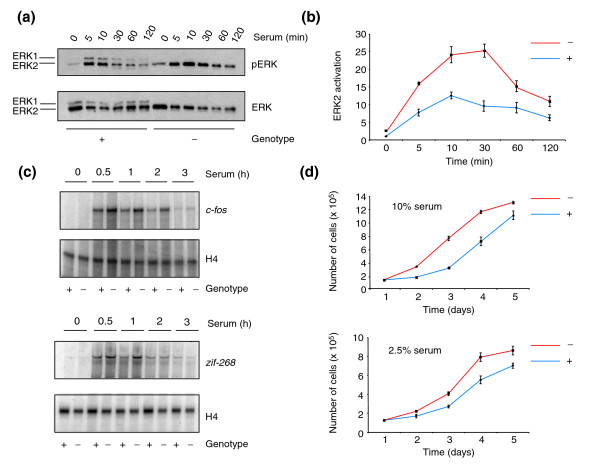
ERK1 ablation in mouse embryo fibroblasts results in enhancement of ERK2 activity and facilitates cell proliferation. **(a) **Wild-type (+) and ERK1-deficient (-) mouse embryonic fibroblasts (MEFs) were serum starved for 24 h and then stimulated with 20% serum for the indicated times. Western blotting was performed with both anti-ERK and anti-phospho-ERK antibodies. **(b) **Bands from (a) were quantified and the fold increase in phospho-ERK2 levels over total ERK2 levels calculated. **(c) **RNA from cells stimulated as in (a) was subjected to an RNase protection assay and probed for either *c-fos *or *zif-268*. A histone H4 probe was used as internal standard for normalization. **(d) **Wild-type (+) and ERK1-deficient (-) MEFs were seeded in triplicate in the presence of either 10% or 2.5% serum and cells were counted after the indicated times. Data are the mean ± standard error of the mean (SEM) of three independent experiments.

We have now also observed that in serum-starved mouse embryo fibroblasts (MEFs) stimulated with 20% serum, ERK2 activation was more sustained in ERK1 mutant cells than in control fibroblasts (Figure 1a). When serum-starved MEFs were stimulated with 20% serum, ERK2 activation was more sustained in ERK1 mutant cells than in control fibroblasts (Figure 1a). Quantification of three independent experiments shows that ERK2 activation is approximately two-fold greater in ERK1 mutant cells than in wild-type cells (Figure 1b). Enhanced ERK2 activation also resulted in increased transcription of immediate-early genes, such as *c-fos *and *zif-268*, as indicated in Figure 1c. As the observed change in ERK2 activation in ERK1 mutant MEFs might have consequences at the level of cell proliferation, we performed a proliferation assay comparing wild-type and ERK1 mutant cells at two different serum concentrations. The results in Figure 1d clearly suggest not only that ERK1 might be dispensable for cell proliferation but also that its absence could provide a significant growth advantage. Together, these data suggest that removal of ERK1 could facilitate ERK2-dependent signaling, cell growth and overall proliferation in MEFs. Importantly, the same results were obtained with MEFs derived from mice either backcrossed to C57Bl/6 background (seven generations) or in a mixed background (C57Bl/6 and 129 SvJ), ruling out a genetic background effect (data not shown).

### Specific knockdowns of ERK1 and ERK2 demonstrate a differential role for the two kinases in cell signaling

**Figure 2:**
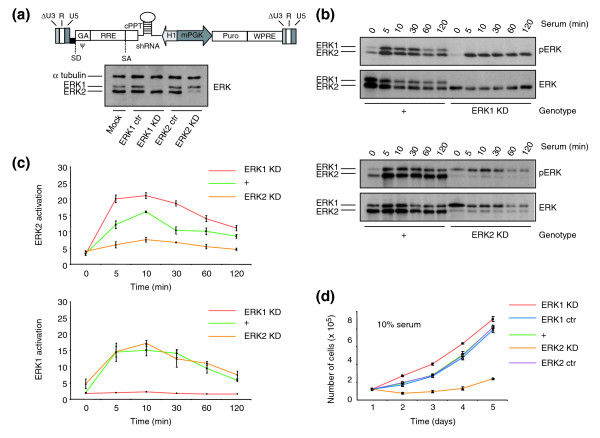
ERK-specific gene silencing unmasks differential roles for ERK1 and ERK2 in cell signaling and proliferation. **(a) **Schematic representation (top) of the proviral vector form used in shRNA-mediated RNA interference. ΔU3, R and U5 constitute a chimeric long terminal repeat (LTR) of the HIV-1 5' LTR with a deletion in U3 abolishing LTR mediated transcription; SD and SA, splice donor and acceptor sites; ψ encapsidation signal including the 5' portion of the *gag *gene (GA); RRE, Rev-response element; cPPT, central polypurine tract; shRNA, small hairpin RNA; H1, human H1 promoter; mPGK, mouse phosphoglycerate kinase promoter; Puro, puromycin-resistance gene; WPRE, woodchuck hepatitis virus post-transcription regulatory element. The western blot (bottom) shows expression levels of ERK proteins in wild-type MEFs transduced with equal amounts of lentiviral vectors carrying the indicated knock-down (KD) shRNA cassette or the corresponding control sequence (ctr). α-tubulin was used as a loading control. **(b) **Wild type (+), ERK1 KD or ERK2 KD MEFs were serum starved for 24 h and then stimulated with 20% serum for 5, 10, 30, 60 and 120 min. Western blots were analyzed with anti-phospho-ERK and anti-ERK antibodies, as in Figure 1. **(c) **Bands from (b) were quantified and fold increases in phospho-ERK2 or phospho-ERK1 levels over total ERK2 or total ERK1 levels calculated. Mean ± SEM of three experiments is indicated. **(d) **Growth curve of wild-type, ERK1 and ERK2 KD fibroblasts and their corresponding controls, seeded in triplicate in the presence of 10% serum and 2 μg/ml puromycin and counted after the indicated times. The data are the mean of three independent experiments ± SEM.

One of the limitations of the global gene-targeting approach is that adaptations over time might occur in the mouse line, possibly producing secondary phenotypes that are not directly linked to the mutation. Therefore, to independently confirm and extend previous findings, we took advantage of RNAi technology by introducing transient knockdowns (KD) of both ERK1 and ERK2 [37-40]. ERK1- and ERK2-specific short hairpin RNAs (shRNAs) were expressed by means of a lentiviral vector (LV) in MEFs under the control of the H1 promoter (Figure 2a). Expression of ERK2 was reduced to less than 10% of the wild-type level, whereas ERK1 became essentially undetectable (Figure 2a). After LV infection and subsequent puromycin-resistance selection, cells were serum starved and subsequently stimulated with 20% serum. As shown in Figure 2b, although ERK1 KD resulted in a significant increase in ERK2 activation profile, loss of ERK2 only marginally affected ERK1 phosphorylation. A quantification and normalization of the data is found in Figure 2c. To determine the consequences of these gene ablation experiments on cell growth we performed growth curves at 10% serum of ERK1 KD and ERK2 KD cells (Figure 2d). Whereas inhibition of ERK2 dramatically reduces cell growth, loss of ERK1 significantly facilitates proliferation. These observations are in accordance with the data obtained in the ERK1^-/-^MEFs (see Figure 1) and further support a potential modulatory role of this kinase in cell-signaling control. Importantly, ablation of ERK2 is sufficient to significantly slow down cell proliferation, a phenotype that strongly resembles the effect of MEK inhibitors such as PD98059 or UO126 ([41,42] and reviewed in [43]).

### Differential MEK-ERK1 and MEK-ERK2 interactions

**Figure 3:**
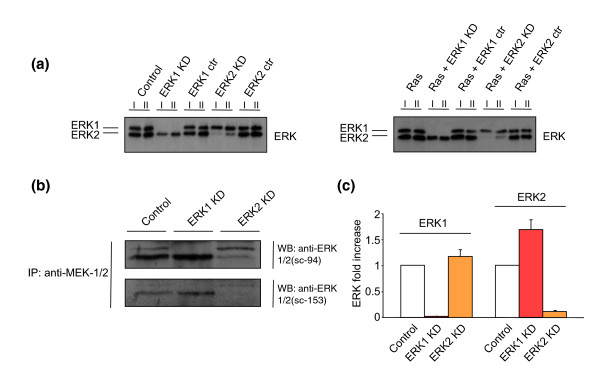
ERK-specific gene silencing in NIH 3T3 cells differentially affects MEK-ERK interactions. **(a) **ERK1- and ERK2-specific NIH 3T3 clones with stable shRNA expression only (left) or also co-transfected with H-Ras^Q61L ^(right) were isolated and checked for ERK expression levels by western blot analysis, as in Figure 1. Two clones (I and II) for each transfection are shown. **(b) **Lysates from wild-type NIH 3T3 control, ERK1 KD and ERK2 KD clones growing in 10% serum were incubated with anti-MEK-1/2 polyclonal antibody. Immune complexes (IP) were resolved in SDS-PAGE and western blotted (WB) with polyclonal anti-ERK1 (sc-94, top) and anti-ERK2 (sc-153, bottom) antibodies. **(c) **Bands from (b) were quantified and the relative fold increase in ERK1 and ERK2 levels in the knockdown samples over the corresponding wild-type controls were calculated (only samples probed with anti-ERK antibody sc-94 are indicated). Data are representative of three independent experiments, expressed as mean ± SEM.

**Figure 4:**
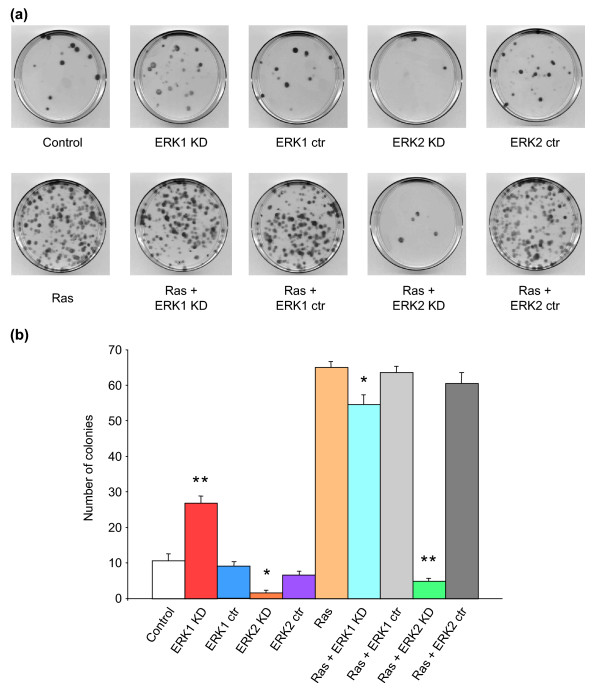
ERK1 knockdown in NIH 3T3 cells facilitates growth in colony formation assays, whereas ERK2 knockdown shows inhibitory effects. NIH 3T3 cells were transfected as indicated with the specific shRNA construct (KD) against ERK1 or ERK2 or the corresponding control sequence (ctr), all cloned into the pSUPER_Puro vector; cells were transfected either with shRNA alone or also with an oncogenic form of H-Ras (Ras^Q61L^), and colony formation was scored after 10 days. **(a) **Representative plates; **(b) **graph of the number of colonies formed (the result of four independent experiments, expressed as mean ± SEM). Asterisks indicate a statistically significant genotype effect calculated from a post-hoc comparison in one-way ANOVA (Scheffe's test: control versus ERK1 KD; control versus ERK2 KD; Ras^Q61L ^versus Ras^Q61L^-ERK1 KD; Ras^Q61L ^versus Ras^Q61L^-ERK2 KD); single asterisk, *p *< 0.01; double asterisk, *p *< 0.0001.

To further explore the molecular mechanisms underlying the observed effects on cell physiology of the two ERK kinases, we generated stable ERK1- and ERK2-specific KD clones in NIH 3T3 cells. As indicated in Figure 3a (left panel), silencing of either ERK1 or ERK2 was as effective in NIH 3T3 cells as in MEFs and did not alter expression of the remaining isoform (Figure 3a, right panel). Moreover, expression of oncogenic H-Ras^Q61L^ had no effect on the protein levels of either ERK1 or ERK2 (Figure 3b, right panel), regardless of the genetic background (wild type, ERK1 KD or ERK2 KD). The latter evidence allowed us to test directly the consequences of ERK-specific gene silencing in a Ras-sensitized background (see below and Figure 4).

One of the assumptions of the competition model is that in activated cells MEK-ERK complexes should preferentially contain ERK2. Moreover, in the absence of ERK1 we would expect to observe a significant increase in MEK-ERK2 interactions. To investigate this possibility and to provide a direct support for the model, we performed immunoprecipitation studies with a specific antibody recognizing both MEK isoforms and then determined the composition of ERK1 and ERK2 in the complex with two distinct antisera. As indicated in Figure 3b, in the absence of ERK1, binding of ERK2 to MEK appears slightly but significantly increased. Quantification of three experiments in Figure 3c demonstrates that ERK2 levels associated with MEK in the absence of ERK1 are 70% higher than in the control extracts. We detected a much smaller change in ERK1 levels in ERK2 KD cells (20%), however. This could possibly be due to a combination of various factors: the presence of some detectable residual ERK2 protein in the MEK complex from ERK2 KD cells; a lower expression level of ERK1 in comparison with ERK2; or a potentially lower affinity of ERK1 for MEK1/2.

### ERK1 knockdown in NIH 3T3 cells facilitates growth, whereas ERK2 knockdown inhibits it

Cell-cycle progression is highly regulated in multicellular organisms, and the loss of any regulatory mechanism could result in tumor formation. Cancer cells can grow in multiple layers and in anchorage-independent conditions, showing less ordered growth and reduced cell-cell contact inhibition. To determine the role of ERK1 and ERK2 in cell growth and Ras-mediated transformation, we made knockdowns of both ERK isoforms in NIH 3T3 cells, either in a wild-type or in oncogenic H-Ras^Q61L ^background, and performed a colony-formation assay, a common test for cell transformation. In this assay, cells transformed with oncogenes such as Ras produce colonies of larger size than cells transfected with vector alone [44]. Importantly, this test does not rely on stable transfectants, as selection and scoring are done within 10 days of transfection.

Representative plates of each transfection are shown in Figure 4a. The summary data shown in Figure 4b clearly indicate that ERK2 knockdown negatively affects both normal and Ras-mediated cell growth. Although loss of ERK1 caused a significant increase in the growth of wild-type cells, however, the effect of the ablation of this MAP kinase on H-Ras^Q61L^-dependent proliferation was surprisingly marginal, and unexpectedly in the direction of a small decrease rather than an increase. These data confirm that ERK1 can negatively modulate normal cell growth in NIH 3T3 cells. It seems, however, that loss of ERK1 in ERK1 KD cells cannot further increase Ras-mediated cell transformation but rather causes a small but consistent reduction in the colonies produced by this potent oncogene. Although this fact suggests that overexpression of oncogenic Ras could determine a ceiling effect in the rate of cell growth, it also leaves open the possibility that in such conditions of abnormally high signaling activation, ERK1 might still have a role qualitatively similar to that of ERK2 and could be positively engaged in the generation of cell-proliferation responses.

### Ectopic expression of ERK1 but not of ERK2 results in the inhibition of Ras-dependent cell growth

**Figure 5:**
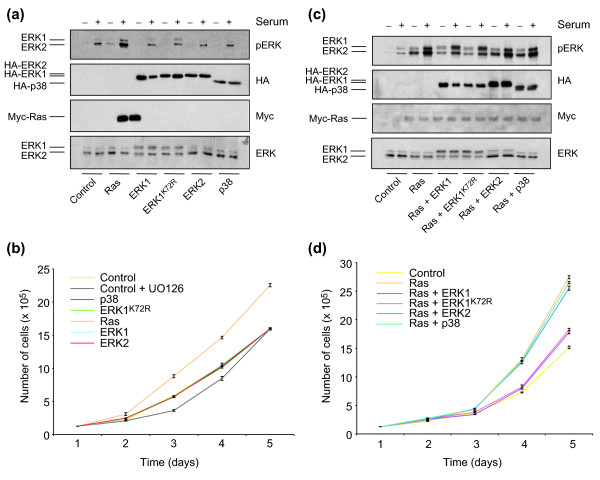
Overexpression of ERK1 attenuates Ras-dependent cell growth in NIH 3T3 cells. **(a) **NIH 3T3 cells were stably transfected with different plasmids bearing hemagglutinin (HA) epitope-tagged ERK1, ERK1^K72R^, ERK2 or p38 or Myc epitope-tagged Ras^Q61L^, all in the vector pMEX. Stable transfectants were generated and expression of the transgene monitored by western blotting. Clones were also serum starved and stimulated with 20% serum for 10 min and extracts were probed with either anti-ERK or anti-phospho-ERK antibodies. **(b) **Three independent NIH 3T3 clones per plasmid from (a) were plated in 10% serum and their growth was monitored for 5 days, as in Figure 1d. The data are the mean ± SEM of three independent experiments. **(c) **Expression of double transfectants was determined as in (a). **(d) **Clones from (c) were monitored for cell growth as in (b). Data are expressed as mean ± SEM of three independent experiments.

To provide further independent and reverse confirmation that the biochemical and proliferation effects observed in the ERK1 mutant MEFs and ERK1 knockdown NIH 3T3 cells are directly linked to the expression level of this protein, we established NIH 3T3 clones individually expressing ERK1, ERK2, ERK1^K72R ^(a kinase-defective form), p38^SAPK1 ^(stress-activated protein kinase, a negative control) and H-Ras^Q61L^, all epitope tagged (Figure 5a). All constructs were expressed at comparable levels. The proliferation profile of three independent clones per genotype is shown in Figure 5b. Ras^Q61L ^provided a significant growth advantage to NIH 3T3 clones, but none of the other constructs alone affected basal levels of cell proliferation. These data suggest that neither ERK1 nor ERK2 overexpression *per se *can alter proliferation of untransformed cells. This is in marked contrast with the RNAi data (see Figure 4) and with the effect of the MEK inhibitor UO126 on the growth of wild-type cells (Figure 5b). Possibly, protein levels achieved with a relative mild level of ectopic ERK1 expression are not sufficient to alter the MEK-ERK2 ratio in the basal state. It is also possible, however, that the effect of ERK1 could be unmasked in deregulated growth conditions, such as in the presence of oncogenic Ras.

Therefore, we next asked whether ectopic expression of these kinases might interfere with growth of transformed cells, by examining the growth of double transfectants containing oncogenic Ras and one of the wild-type or mutant kinases described above. The underlying idea, taken from previous genetic studies in *Drosophila *and in mouse, was that the possible effect of a 'modulator' of cell growth might be manifested in a sensitized background, here provided by Ras^Q61L^. Surprisingly, expression of ERK1 kinase resulted in a significant reversion of the cell-proliferation effect caused by oncogenic Ras, whereas neither ERK2 nor p38 seemed to affect the overall growth rate (Figure 5c,d).

These data indicate that ERK1 protein expression counteracts Ras-dependent cell transformation. Importantly, this effect seems to be largely independent of ERK1's kinase activity but rather is due to protein-protein interactions, as the ERK1^K72R ^mutant was almost as effective as wild-type ERK1. Strikingly, overexpression of ERK2 has little effect on cell proliferation, suggesting that levels of this protein are not rate-limiting, at least in this cell type.

### Ectopic expression of ERK1 attenuates Ras-dependent growth in transformation assays

**Figure 6:**
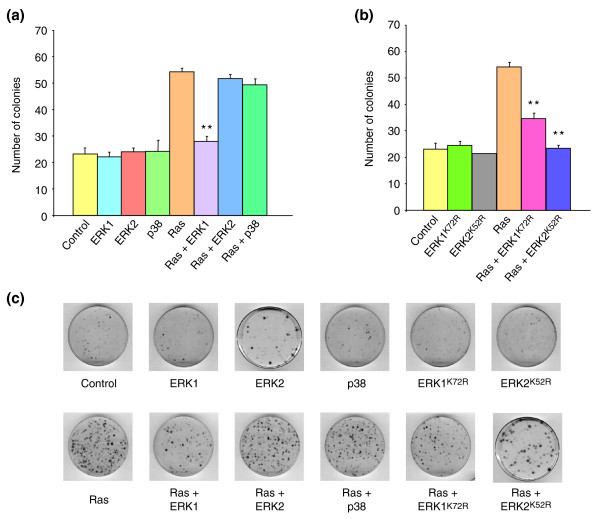
Ectopic expression of ERK1 in NIH 3T3 cells inhibits Ras-mediated colony formation. **(a,b) **NIH 3T3 cells were transfected as indicated and colony formation was scored after 10 days. Graphs represent quantitations of six independent experiments, expressed as mean ± SEM. Double asterisk indicates a genotype effect that is statistically significant (*p *< 0.0001), calculated from a post-hoc comparison in one-way ANOVA (Scheffe's test: Ras^Q61L^-ERK1 versus Ras^Q61L^-pMex; Ras^Q61L^-ERK1^K72R ^versus Ras^Q61L^-pMex; Ras^Q61L^-ERK2^K52R ^versus Ras^Q61L^-pMex). **(c) **A representative plate for each clone from (a,b) is shown.

To further confirm the role of ERK1 and ERK2 in Ras-dependent cell transformation, we transiently transfected both ERK isoforms into NIH 3T3 cells and performed colony formation assays. Oncogenic Ras (Ras^Q61L^) was co-transfected into NIH 3T3 cells with a control vector (pMEX) alone or with a vector containing either ERK1, ERK2, or p38. Summary results after 10 days are shown and quantitated in Figure 6a. Ras^Q61L ^alone induced a greater number of large colonies than the control vector, whereas ERK1, ERK2 and p38 alone did not differ from the control, indicating that simple ectopic expression of these kinases is not sufficient to change the proliferation rate of NIH 3T3 cells. When co-transfected with Ras^Q61L^, however, ERK1 induced a significant reduction in the number of large colonies compared with Ras^Q61L^, whereas wild-type ERK2 and p38 co-transfected with Ras^Q61L ^had little effect. Representative plates are shown in Figure 6c.

As observed in Figure 5, expression of a kinase-defective mutant of ERK1 was found to be very effective in inhibiting cell proliferation in NIH 3T3. This observation is consistent with the MEK-ERK competition model, as one of the predictions of this model is that a kinase-defective form of ERK1 should efficiently displace the endogenous ERK2 protein from MEK and therefore significantly reduce the overall signaling output. We also speculated, however, that a kinase-defective mutant of ERK2 should act as inhibitor of endogenous ERK2 and that its effect could possibly be even more pronounced than that caused by ERK1. To test this hypothesis we generated a kinase-dead ERK2 mutant, ERK2^K52R^, and compared its effect in the colony formation assay with that of the ERK1^K72R ^mutant [45]. As shown in Figure 6b, both ERK kinase-defective mutants were very effective in reducing oncogenic Ras-mediated colony formation, but ERK2^K52R ^caused an almost complete inhibition whereas ERK1^K72R ^reduced growth to only 40% of the total.

These data further support the idea that ERK1 and ERK2 compete for binding to MEK and therefore that their level of expression is crucial to the fine tuning of output signaling. Importantly, similar results were obtained with a different *in vitro *proliferation test, the soft agar assay (data not shown).

### ERK1 attenuates Ras-dependent tumor formation in nude mice

**Figure 7:**
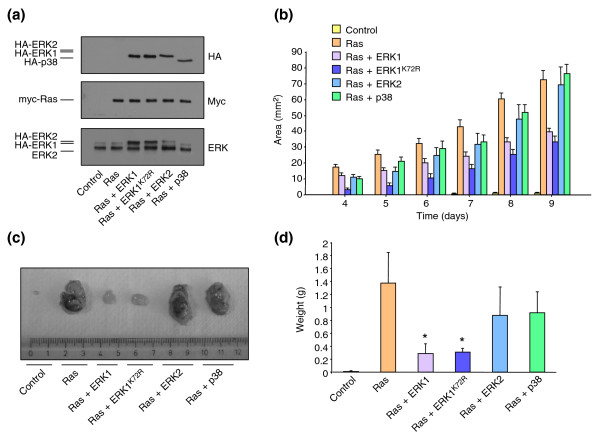
ERK1 expression inhibits Ras-dependent tumor formation in nude mice. **(a) **NIH 3T3 clones were transfected as indicated and expression of the relevant transgenes assessed by western blotting. **(b) **Growth of tumors in injected nude mice was monitored over 6 days starting from day 4 after injection, by determining the skin area covered by the tumor mass (mm^2^). The data are expressed as mean ± SEM of two independent experiments (ten animals per clone). **(c) **Representative tumors after sacrifice at day 10 are shown. **(d) **Mean weight (± SEM) of the different tumor samples is indicated. Asterisk indicates a genotype effect significant at *p *< 0.001, calculated using a post-hoc comparison in one-way ANOVA (Scheffe's test: Ras^Q61L^-ERK1 versus Ras^Q61L^-pMex; Ras^Q61L^-ERK1^K72R ^versus Ras^Q61L^-pMex).

NIH 3T3 cells normally show low tumorigenicity, but when transfected with an oncogene such as Ras^Q61L^, they acquire the ability to induce tumor formation in immunodeficient, athymic mice (nude mice) [46]. We therefore decided to perform a tumorigenicity assay to test the ability of ERK1 to reduce cellular transformation and tumor formation *in vivo*. NIH 3T3 clones stably transfected with Ras^Q61L ^or ERK1, ERK1^K72R^, ERK2 or p38 were tested for transgenic expression and subsequently used in the assay (Figure 7a).

To study tumor growth we used male, 4- to 6-week-old athymic nude mice. Cells of each clone were injected subcutaneously into each flank of the nude mice, using five animals for each clone. Nude mice were examined daily and tumor size was recorded from day 4 to 9, as indicated in Figure 7b. Although both Ras^Q61L^-transformed cells and cells double-transfected with ERK2 or p38 produced large tumors, cells double-transfected with either ERK1 or ERK1^K72R ^produced significantly smaller ones.

The experiment was terminated 10 days after injection and tumors were removed and weighed. Representative explant sizes are shown in Figure 7c and the mean weight data of two experiments are shown in the Figure 7d. Although mice injected with control vector cells did not develop tumors, all the other animals did. Tumors from mice injected with NIH 3T3 cells cotransfected with Ras^Q61L ^and ERK1 or ERK1^K72R ^were, however, significantly smaller (means of 0.32 g and 0.29 g, respectively) than those obtained from mice injected with Ras^Q61L ^(1.2 g, *p *< 0.001 for both comparisons). No significant differences were seen when instead comparing tumors from mice injected with Ras^Q61L ^alone and double transfectants with ERK2 or p38 (1.04 g and 0.98 g on average, respectively).

These data on tumorigenicity *in vivo *fully confirm the results of the *in vitro *functional assay, indicating a potential anti-oncogenic role of ERK1 in its ability to reduce Ras-mediated cell transformation and tumor formation, at least in this experimental system.

**Figure 8:**
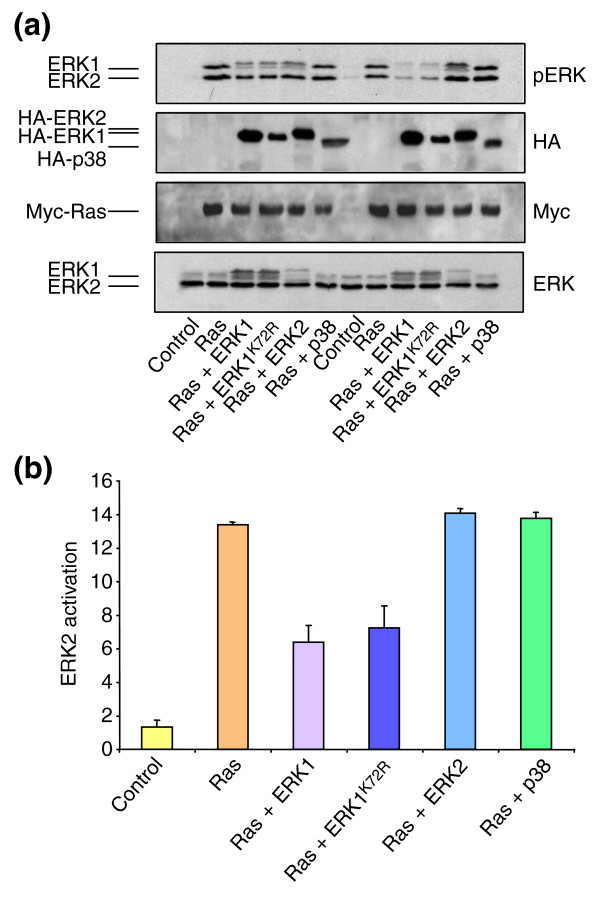
ERK2 activation is significantly reduced in tumors overexpressing ERK1. **(a) **Two individual tumors for each clone were explanted from treated mice and subjected to western blot analysis to determine ERK2 activation and transgene expression. **(b) **Mean ± SEM of the data in (a) are plotted as indicated.

As a final test, we analyzed biopsies from the nude mice by western blot to confirm that a clear correlation could be demonstrated between abnormal growth and ERK2 activation in those tumors (Figure 8a,b). As expected, endogenous ERK2 phosphorylation was greatly upregulated in Ras^Q61L ^samples and double transfectants with ERK2 and p38. Double transfectants with either ERK1 or ERK1^K72R ^showed significantly less pronounced ERK2 activation, however, suggesting a direct link to their carcinogenic potential.

## Conclusion

The recent generation of mice with targeted mutations of the *ERK1 *and *ERK2 *genes has provided an excellent opportunity to determine the level of redundancy and overlap in function between these two major MAP kinase isoforms. ERK1 mutant mice have a strikingly milder phenotype than ERK2 mutants, which die early in development [28,30-32]. At the molecular level, one of the most intriguing features of ERK1-deficient cells is the enhanced stimulus-dependent activation of ERK2 in the absence of any compensatory increase in ERK2 protein levels. We believe that this effect is largely due to a competition between ERK1 and ERK2 in binding to the upstream kinase MEK. In a previous report [33] we supported this claim by showing that the kinase-defective ERK1^K72R ^mutant is just as effective as wild-type ERK1 in rescuing the phenotype in ERK1-deficient MEFs [33]. The aim of the present work was to extend those preliminary findings and explore the possibility that ERK1 acts, at least in certain cellular settings, as a negative modulator of cell proliferation, by interfering with Ras-ERK2-dependent signaling. First we examined growth rates of MEFs obtained from ERK1-deficient animals. In the absence of ERK1, MEFs proliferate faster than control cells. Accordingly, whereas ERK1 knockdown by RNAi in both MEFs and NIH 3T3 cells facilitates cell growth, ERK2 silencing causes severe cell-proliferation deficits. By contrast, a mild over-expression of ERK1 is sufficient to slow down cell growth mediated by oncogenic Ras, and this concomitantly impairs ERK2 activation. This inhibitory effect of ERK1, which cannot be achieved by ectopically expressing ERK2, also affects the ability of oncogenic Ras to transform NIH 3T3 cells in functional assays such as colony formation and to cause tumors in nude mice. ERK1 seems to act by displacing ERK2 from the upstream kinase MEK rather than by regulating downstream effectors, as a kinase-inactive form of ERK1 is equally effective in causing the observed phenotypes. This hypothesis is also directly supported by the observation that in ERK1-deficient fibroblasts, the amount of MEK-ERK2 complexes is significantly increased.

The hypothesis that signaling molecules can act as titrating agents, sequestering central components of the signaling machinery, has already gained some experimental support. For instance, Rap1, a Ras-related small GTPase, was originally identified as a suppressor of K-Ras-mediated transformation because when overexpressed in NIH 3T3 cells it binds with high affinity to Raf-1 and therefore traps this downstream Ras effector in an inactive complex ([47] and reviewed in [48,49]). Moreover, a provocative paper suggested in 2001 [46] that wild-type K-ras protein could itself act as a tumor suppressor of oncogenic K-Ras. In addition, certain B-Raf kinase-defective mutants can be highly oncogenic because they interact with wild-type c-Raf-1 and thus activate MEKs with greater activity [25].

Our observation that ERK1 can interfere with ERK2-dependent signaling follows on from these pieces of evidence, but a few specific features of our results should be highlighted. First, no mutation in the ERK1 protein seems to be required to exert the effect on ERK2 phosphorylation, as can be seen by simply overexpressing wild-type ERK1. Second, no enzymatic activity is strictly necessary, as indicated by the effect of ERK1^K72R^. Importantly, the homologous kinase-dead mutant of ERK2, ERK2^K52R^, is even more effective in interfering with endogenous ERK signaling, further supporting the competition model. Third, complete ablation of ERK1 from cells is sufficient to provide a significant growth advantage, in both primary fibroblasts and cultured NIH 3T3 cells, although this manipulation is ineffective in promoting oncogenic Ras-mediated transformation. Fourth, overexpression of ERK1 does not affect normal growth but only that dependent on oncogenic Ras. All these observations need to be explained by a detailed cellular and biochemical model of ERK regulation, which goes beyond the present work.

Certainly, other regulatory mechanisms are likely to be in place and to contribute significantly to the functional differences between ERK1 and ERK2. For instance, the rate of translocation, dephosphorylation and sequestration in the nucleus of the two ERK proteins could be different. Although our experiments with the two kinase-defective mutants suggest that protein-protein interactions in the absence of kinase activity are dominant in the process, it is formally possible that smaller differences in substrate specificity could also partially contribute to specific aspects of cell physiology controlled by the two isoforms. Another important aspect that should be taken into consideration in the future is the potential role of scaffolding complexes in the differential regulation of ERK1, ERK2 and MEK interactions (reviewed in [50,51]). This possibility was suggested previously by computational studies revealing that an optimal concentration of scaffold proteins relative to their kinase partners is always required to maximize signaling output. Alterations of the ratio of ERK1/ERK2 to MEK and to any scaffold protein within the cell could therefore affect the threshold properties of the system and contribute to some of the non-linear cellular responses [52,53].

Despite these open questions and the limitations of our experimental approach, we believe we have at least identified a new, possibly important aspect of ERK regulation. We are just starting to uncover a system of previously unappreciated complexity, involving dynamic interactions of MEK1/2, ERK1 and ERK2. Future work at the biophysical and computational level will certainly be required to clarify these important mechanisms.

## Materials and methods

### Plasmid construction

To generate plasmids for stable RNAi, oligonucleotides encoding shRNAs against ERK1, ERK2 and related scrambled control sequences were purchased from VBC Genomics (Vienna, Austria), annealed and cloned into *Bgl*II and *Xho*I sites under the control of a constitutive human H1 promoter (pSUPER_Puro) [37]. A set of six 19-mer shRNAs for each MAP kinase gene was designed as described [54] and tested, and the most efficient ones were chosen. The following oligonucleotides were used (bold text indicates the targeted sequence in the coding region that creates the stem of each shRNA; underlined text indicates the point mutations): for mouse ERK1, 5'-GATCCCC**GACCGGATGTTAACCTTCA**TTCAAGAGA**TGAAGGTTAACACCGGTC**TTTTTGGAAA-3' and 5'-AGCTTTTCCAAAAA**GACCGGATGTTAACCTTCA**TCTCTTGAA**TGAAGGTTAACATCCGGTC**GGG-3', targeting exon 7; for mouse ERK2, 5'-GATCCCC**GTACAGAGCTCCAGAAATT**TTCAAGAGA**AATTTCTGGAGCTCTGTAC**TTTTTGGAAA-3' and 5'-AGCTTTTCCAAAAA**GTACAGAGCTCCAGAAATT**TCTCTTGAA**AATTTCTGGAGCTCTGTAC**GGG-3', targeting exon 4; for the ERK1 control, 5'-GATCCCC**GACCGGATAGTAACCTTCA**TTCAAGAGA**TGAAGGTTACTATCCGGTC**TTTTTGGAAC-3' and 5'-TCGAGTTCCAAAAA**GACCGGATAGTAACCTTCA**TCTCTTGAA**TGAAGGTTACTATCCGGTC**GGG-3'; for the ERK2 control, 5'-GATCCCC**GTACAGAGCGTCAGAAATT**TTCAAGAGA**AATTTCTGACGCTCTGTACT**TTTTGGAAC-3' and 5'-TCGAGTTCCAAAAA**GTACAGAGCGTCAGAAATT**TCTCTTGAA**AATTTCTGACGCTCTGTAC**GGG-3'.

All lentiviral constructs were built from plasmid pCCLsin.cPPT.PGK.eGFP.WPRE, as described elsewhere [55]. For cloning into LVs, each shRNA-H1/mPGK-Puro cassette was cut out from pSUPER_Puro vectors and cloned into the *Bam*HI/*Cla*I site of the vector. Enhanced green fluorescent protein (eGFP) was removed by *Bam*HI/*Sal*I digestion, then the vector was blunted and self-ligated.

For ectopic expression in the colony formation assay, mouse ERK1, ERK1^K72R^, ERK2 and ERK2^K52R ^were epitope-tagged at the amino terminus with hemagglutinin and cloned into the pMex-Neo^R ^expression vector. An oncogenic form of H-Ras (H-Ras^Q61L^) was instead Myc-tagged and also cloned into the pMex-Neo^R ^expression vector.

### Viral vector production and titration

VSV-pseudotyped third-generation LVs were produced by transient four-plasmid cotransfection into 293T cells using the calcium phosphate method and purified by ultracentrifugation, with the modification that 1 mM sodium butyrate was added to the cultures for vector collection. Supernatants were collected 48 h after transfection, filtered through a 0.22 mm membrane and concentrated by ultracentrifugation as described [56].

The average number of viral vector particles were measured by the HIV-1 gag p24 antigen immunocapture assay (NEN Life Science Products, Boston, USA) and viral titer was determined by real-time PCR [57].

### Cell culture and biochemistry

MEF cultures were prepared from wild-type and knockout embryos at embryonic day 13.5. Cells at early passages were serum starved for 24 h and then stimulated with 20% serum for various times; protein was then extracted and analyzed by SDS-PAGE and western blotting [33]. For the RNase protection assay, RNA extraction and analysis from stimulated cell monolayer was performed as previously described [58]. For cell-proliferation studies in MEFs or NIH 3T3 cells, 1.25 × 10^5 ^cells were seeded in each six-well plate on day 1 and maintained in Dulbecco's Modified Eagle Medium (DMEM) at different serum concentrations, with 2 μg/ml puromycin if required. Medium was changed every day. Duplicate samples for each serum concentration and for each genotype were counted every day for 5 days using a Burker counting chamber. For biochemistry, NIH 3T3 cells were transfected with pSUPER_Puro-shRNAs or with hemagglutinin epitope-tagged ERK1, ERK1^K72R^, ERK2, p38 and Myc epitope-tagged H-Ras^Q61L ^cloned into the pMex-Neo^R ^vector by calcium phosphate precipitation [59]. Stable transfectants were obtained after selection in G418 (Geneticin, Gibco-Invitrogen, Carlsbad, USA) or puromycin (Clontech, Mountain View, USA).

### Immunoprecipitation assay

NIH 3T3 cells were stably transfected with ERK1 and ERK2 knockdown or control plasmids. Cells were washed with ice-cold phosphate-buffered saline before extraction in 1 ml of lysis buffer (20 mM Tris pH 7.5, 150 mM NaCl, 10% glycerol, 5 mM EGTA, 1 mM EDTA, 1% Triton X-100, 2 mM MgCl_2_, 50 mM NaF, 10 μM Na_3_VO_4_) containing 0.2 mM phenyl methyl sulfonyl fluoride (PMSF) and 1× COMPLETE cocktail of protein inhibitors (Roche, Basel, Switzerland). Extracts were clarified at 14,000 *g *for 5 min at 4°C. Equal amounts of each protein lysate (5 mg) were pre-cleared with ExtraCruz-F immunoprecipitation (IP) matrix (Santa Cruz Biotechnology, Santa Cruz, USA) for 30 min at 4°C and incubated for an additional 1 h with polyclonal anti-MEK-1/2 antibodies (sc-436, Santa Cruz) complexed with the IP matrix. Immune complexes were collected and washed three times with lysis buffer, and western blotting was performed with polyclonal anti-ERK1 (sc-94) and anti-ERK2 (sc-153) antibodies (Santa Cruz), which preferentially recognize ERK1 and ERK2, respectively.

### Colony formation assay in NIH 3T3 cells

Colony formation assays were performed as previously described [44]. On day 0, NIH 3T3 cells were seeded in 100-mm plates, 1.5 × 10^5 ^cells per plate, and were transfected the following day. Two days after transfection, cells were trypsinized and plated on 100-mm plates, 10^3 ^cells per plate in DMEM containing 10% bovine calf serum and 500 mg/ml G418 for the selection of neomycin-resistant cells. Each transfection sample was plated into four plates. Medium was changed every 3 days and after 10 days clones were washed with water and fixed in 10% formaldehyde (Sigma-Aldrich, St. Louis, USA) for 10 min. Plates were then washed once with water and stained for 5 min in 0.5% crystal violet (Fluka, Sigma-Aldrich, St. Louis, USA) in 20% methanol and finally washed with water to remove background staining. Images were acquired with a scanner and all colonies larger then 1.5 mm in diameter were counted.

### Tumorigenicity assay in nude mice

G418-positive clones selected as described above were used for the tumorigenicity assay [46]. 1 × 10^6 ^cells of each clone in log-phase growth were injected subcutaneously into each of two flanks of male 4- to 6-week-old athymic nude mice. For each clone five animals were used. The nude mice were examined daily and tumor sizes were recorded. The experiment was terminated 10 days after injection, animals were sacrificed and tumors were removed and weighed.
